# Analysis of the impact of deep learning know-how and data in modelling neonatal EEG

**DOI:** 10.1038/s41598-024-78979-y

**Published:** 2024-11-14

**Authors:** Aengus Daly, Gordon Lightbody, Andriy Temko

**Affiliations:** 1 Department of Mathematics, Munster Technological University, Cork, Ireland; 2 Department of Electrical and Electronic Engineering, University College Cork, Cork, Ireland; 3 INFANT Research Centre, University College Cork, Cork, Ireland

**Keywords:** Biomedical engineering, Neonatal brain damage

## Abstract

The performance gains achieved by deep learning models nowadays are mainly attributed to the usage of ever larger datasets. In this study, we present and contrast the performance gains that can be achieved via accessing larger high-quality datasets versus the gains that can be achieved from harnessing the latest deep learning architectural and training advances. Modelling neonatal EEG is particularly affected by the lack of publicly available large datasets. It is shown that greater performance gains can be achieved from harnessing the latest deep learning advances than using a larger training dataset when adopting AUC as a metric, whereas using AUC90 or AUC-PR as metrics greater performance gains are achieved from using a larger dataset than harnessing the latest deep learning advances. In all scenarios the best performance is obtained by combining both deep learning advances and larger datasets. A novel developed architecture is presented that outperforms the current state-of-the-art model for the task of neonatal seizure detection. A novel method to fine-tune the presented model towards site-specific settings based on pseudo labelling is also outlined. The code and the weights of the model are made publicly available for benchmarking future model performances for neonatal seizure detection.

**Supplementary Information:**

The online version contains supplementary material available at 10.1038/s41598-024-78979-y.

## Introduction

In recent years deep learning has emerged as the most successful machine learning (ML) method in human perceptual-based tasks, excelling in image, audio/speech and text recognition^1–5^. This success of deep learning comes from its ability to take advantage of both increasingly larger datasets and more complex architectures and methodologies^4,6,7^. This success has been further aided by advancements in computing hardware such as GPUs, TPUs, as well as well-developed publicly available software infrastructures like Pytorch and Tensorflow.

In many applied ML domain areas, such as diagnosis using medical images or seizure detection using electroencephalography (EEG) signals for example, the collection of such large datasets required for deep learning can be challenging. The process of annotation of these datasets requires detailed analysis by highly trained experts which is laborious, expensive and error prone^8,9^. There may also be ethical and regulatory barriers to overcome before such datasets can be used. Researchers in these areas can often lack deep understanding of ML methodologies and falsely believe that data is the only thing that is required to succeed^10^, thus concentrating more effort on data accumulation than technical innovation. In contrast, the ImageNet dataset^5^, with over 14 million annotated images, is a prime example where the public availability of high quality data has enabled performance increases in the area of computer vision through genuine architectural innovations and new enhanced optimization routines in deep learning.

Analysis of neonatal EEG is an example of an applied ML area where data scarcity is pronounced; publicly available neonatal EEG databases are very limited in size^11,12^. The early detection of neonatal seizure events is crucial for subsequent cognitive and physiological development of newborns. The primary method of detecting a seizure is to analyse brain wave activity via EEG. While machines that monitor and record EEG are widely available in most developed countries, the availability of experts that can interpret these signals is limited^13^. In recent years, this urgent clinical need has attracted research to develop ML-based decision support systems to assist clinicians in detecting seizures. Early works in seizure detection using EEG utilized rule-based approaches that mimicked experts’ visual inspection methods and progressed to include methods that required expertise in EEG signal processing^14–16^. As more data was collected, these were replaced by more data-driven approaches, firstly with ML methods, which required expertise in feature engineering from EEG signals^17–20^. More recently deep learning methods have become popular^21,22^ with less emphasis placed on feature engineering and more on expertise in deep learning architectures and training routines.

Neural networks using sequential convolutional layers (ConvNets), the primary component of many backbones in computer vision, have also become the main ingredient of deep learning models in neonatal seizure detection using EEG^23–26^. ConvNets’ dominance is due to their high performance and adaptability which are mainly attributed to their translation invariance capabilities, computational efficiency and their ability to take as input a sliding window series of data^3^. These adaptable attributes enable ConvNets to be harnessed by other applications and domain areas that employ visual feature learning, such as seizure detection which use as inputs EEG signals as image-like representations.

Recent works in neonatal seizure detection using deep learning that take as inputs raw multi-channel EEG signals include a fully convolutional ConvNet^23^, which is detailed in Sect. 2.1 below, a ConvNet using L2 regularization and drop-out^25^ and a ConvNet using the 3 different annotators of a dataset to build an ensemble of 3 separate models^24^. Another study used a ConvNet as a feature extractor for a variety of ML classifiers, with Random Forest giving the best performance^27^. A graph based ConvNet has also been used where EEG signals were initially converted to graph representations that were subsequently fed into a graph ConvNet classification model^28^. In a later work these researchers developed this model further by adopting attention mechanisms which can help in highlighting the potential seizure location^29^. Data augmentation techniques were utilized in another study where the amplitude of the EEG signal was randomly altered along with residual based connections in a fully convolutional ConvNet model^30^. Another study compared 2 ConvNet based models, one with Inception layers that applied a variety of filter sizes to the same layers of the network and a second model that inputs the representations generated from first model into an attention based mechanism, which gives more weight to the EEG channels that indicate seizure characteristics^31^. Transfer learning via the well-established models AlexNet, ResNet18, GoogleNet, DenseNet and ResNet50 have also been used^32^, models that were pretrained using the ImageNet dataset^5^ and originally developed in the computer vision domain. This work also used as input colour image representations of the EEG signal. A recently published work presented a novel approach through the use of the sonification of the EEG signal in conjunction with a fully convolutional sequential ConvNet model^33^. These studies use different clinical datasets of various sizes making it difficult to compare them and further to understand whether the improvement in performance has been due to technical novelties or larger/better annotated/more diverse training or test datasets.

Developments in deep learning techniques and architectures in domains that utilize visual feature learning, such as neonatal seizure detection, draw much of their inspiration from innovations in the computer vision arena where the architectural inductive bias has been shifting from one to another over a period of several years, from classic sequential ConvNets^34^ to Recurrent Neural Networks (RNNs)^35^ to Transformers which use attention mechanisms^36^ to hybrid models^37^ and back to more advanced ConvNets^3^. It has been recently shown that by incorporating the latest advances in deep learning methods and training regimes these advanced ConvNets provide state-of-the-art performance levels for a variety of image recognition tasks^3^. In this research we investigate adopting these latest methods for the task of neonatal seizure detection. This study investigates how much performance can be extracted from the only publicly available dataset in this area using these latest methods. Using this publicly available dataset as a baseline training dataset this study aims to contrast the amount of improvement achievable from having access to a larger training dataset versus the amount of improvement one can get from leveraging the latest advancements in deep learning technical know-hows.

The main contributions of this study are:

- A baseline and enhanced deep learning architectures are trained both on a small dataset and larger dataset to analyse whether having more data is more beneficial than architectural and training innovations.
- An enhanced deep learning model with architectural and training advancements is presented and its performance is compared to the state-of-the-art results of a baseline model, the current publicly available benchmark as well as a commercially available tool using a variety of metrics.
- The code and weights of the new state-of-the-art model are made publicly available to establish the new benchmark for neonatal seizure detection and are available at https://github.com/CiallC/Neonatal_seizure_detection_dl_algorithm.
- A novel process based on pseudo-labelling is presented on how to adapt the released model to site-specific settings with a small sample of unlabelled data of the targeted deployment site.

## Methods

Three previously developed methods for neonatal seizure detection from EEG are used in this study for comparison – a baseline employing a fully convolutional architecture^23^, a publicly available system based on the SVM model^20^, and a commercially available system based on the SVM model^17^. An enhanced deep learning model for neonatal seizure detection that incorporates many of the latest technical advances is proposed and reviewed.

### Baseline deep learning model

A novel fully-convolutional deep learning architecture was developed in 2019^23^. The main distinctive components of the developed system were the use of:

- Multichannel raw EEG as an input. No hand-crafted feature engineering was required, the features were learned on the fly from raw multichannel EEG data with deep sample-size filters. The system can absorb any number of input EEG channels. Without hand-engineered features the network was optimized end-to-end.
- A fully convolutional architecture. The absence of dense layers allows the network to be independent of the length of the input and adds an element of regularization. Further as the temporal relationship between the input and the output is preserved throughout the transformation detection and localisation of highly impactful EEG segments in time can be performed.
- Weak labels for training. It was the first system that could be trained on weakly labelled data. The weakly labelled data refers to the recordings that were clinically labelled in time as seizure or non-seizure segments without specifying the EEG channels on which seizures could be seen. This utilization of weak labels massively increased the amount of data that can be used for training.

The Baseline model generates features using 3 identical stacked feature extraction blocks (FEBs). Each FEB is composed of 3 convolutional layers of 32 filters of sample-size 3, followed by an average pooling layer. These 3 FEBs feed into a classification block (CB) which consists of a convolution layer and a global average pooling layer. This model was trained using the parameters as outlined by its developers^23^,namely a batch size of 300 with batch normalisation and stochastic gradient descent, with a learning rate of 0.001 and Nesterov momentum of 0.9 and the glorot uniform random weight initializer. The training routine we used is the same as we used for the enhanced model, detailed in Sect. 2.4.1. below.

This model incorporated three postprocessing steps that aim to reduce the number of false seizure detections, previously developed by the co-authors^19^. First, a central non-causal moving average filter (MAF) of 60s duration is applied to smooth the probabilistic output. This is followed by a per-patient adaptive adjustment for the level of background probabilistic activity by contrasting the current probability with the average of the previous 10 min. Finally, a detected seizure event is extended on both sides by a ‘collar’ of 30s to compensate for the delay introduced by moving average. This collar introduces a 30s latency in the real time detection of seizures which the authors believe is acceptable in a clinical setting.

### Public SVM model

An SVM-based algorithm to detect neonatal seizures was developed in 2019^20^. It was trained on the publicly available dataset^11^. The system uses 21 hand-crafted features as inputs to an SVM model. These hand-crafted features are engineered from EEG signals and are created from time and time-frequency representations of the EEG signal. The code for this algorithm is publicly available^38^ and during our experiments we used the default parameters. The main aspects of this model were:

- Incorporation of engineered features that capture non-stationary correlation in the time-varying periodicity of neonatal EEG.
  - Features from spike correlations from adaptively extracted segments of the EEG signal.
  - Features from time-frequency correlations between scale shift time-slices of a time-frequency distribution.
- 19 additional hand engineered features derived from these and the power of the EEG signal were added to form a detection statistic.
- A postprocessing ‘collar’ of 23s is applied to take account of the overlap in an epoch segmentation technique and is a parameter that was optimised by the developers of this SVM. Detected seizures are extended forward in time by 23s which introduces a latency in the system.

### Commercial SVM model

Another SVM-based system was developed^17^ and enhanced in 2013 by the co-authors^19^. This algorithm has been clinically trialled and adopted in a clinical setting across several European Hospitals^39–41^. The algorithm relies on a set of 55 hand-engineered feature to capture temporal, frequency, information-theory and structural information in the neonatal EEG signal. This algorithm is not publicly available but was available to the authors. Extensive details have been published^17,19^. The main attributes of this model were:

- Utilization of a large and diverse hand-crafted feature set to represent each EEG channel separately. The model can run on any number of EEG channels available.
- The novel postprocessing that performed smoothing of the decisions in time in the probabilistic domain. These same postprocessing steps, detailed above in Sect. 2.1, were utilized in the Baseline model and in the Enhanced model.
- A novel background adaptive scheme to compensate for variable levels of respiration artefact and background noise in the data.

### Enhanced model

As previously mentioned, recently it has been shown that by harnessing the latest developments in deep learning training methods ConvNets produce state-of-the-art performance levels for a range of image recognition tasks^3^. The significance of adopting these key architectural and training methodological advances is analysed in this research and compared to significance of harnessing a larger training dataset for the task of neonatal EEG modelling.

**Fig. 1:**
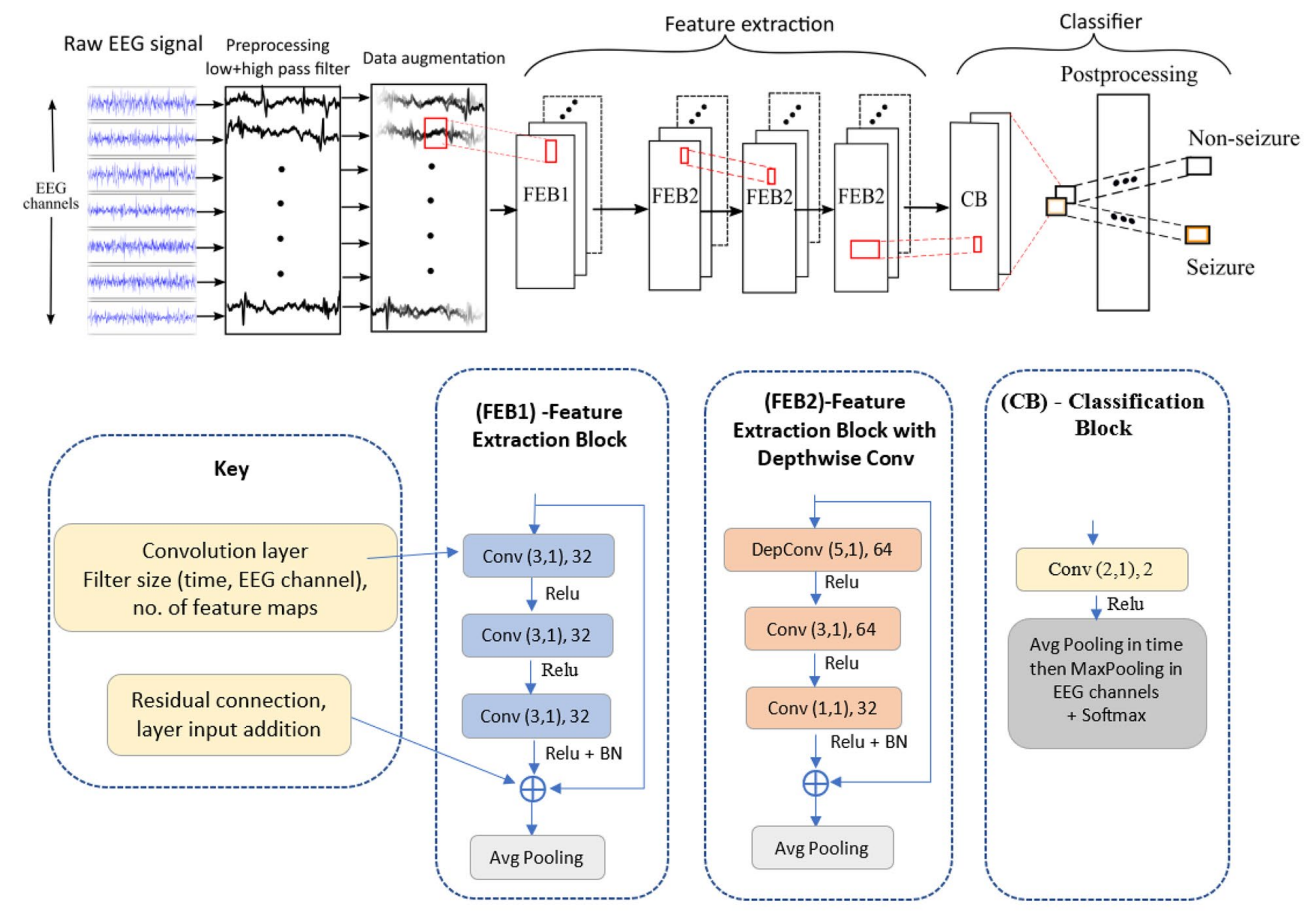
The Enhanced model takes as input raw EEG signal which is preprocessed and augmented. Features are generated from this using 4 Feature Extraction Blocks (FEBs) which comprise of convolutional layers, an average pooling layer and residual connections, details are given in the ‘Key’ above. FEB2 begins with a Depthwise convolutional layer with 64 filters of 5 sample size. The ReLU activation function is used and Batch Normalisation (BN). The features are fed into a classification block (CB), followed by postprocessing steps, classifying the original input of EEG signal as either seizure or non-seizure. In total there are 45 K trainable parameters.

The enhanced architecture proposed here is structurally based on the fully convolutional architecture from the Baseline model. The information characteristics of neonatal seizures is known to be predominately found in the range between 0.5 and 12 Hz^42^. Thus the EEG signal is pre-processed with a high and low pass filter of 0.5 Hz and 12.8 Hz respectively, to reduce noise and unwanted artefacts, and then down-sampled to 32 Hz. A 16s sliding window of this pre-processed EEG signal is input into the model with a 1s shift. While this input is two-dimensional the convolutional and the pooling layers are essentially one-dimensional, operating along the time axis. The postprocessing techniques developed in the commercial SVM model and detailed above in Sect. 2.1 are also used here. The enhanced pipeline is shown in Fig. 1. The main distinctive features of the Enhanced model are:

- Architectural – while the model employs a fully convolutional model, it is enhanced with residual connections^43^, depthwise convolutions, bottleneck layers, a larger receptive field and a variety of kernel sizes.
- Data augmentation – two data augmentations techniques are exploited during training, namely dynamic amplitude rescaling and Mixup^44^. Both data augmentation techniques add an element of signal diversity and a small random amount of noise to the training dataset, thus acting as an additional regularizer to help prevent overfitting.
- Optimization – the RAdam optimizer^45^ is employed which utilizes a warm-up heuristic to control the variance of the adaptively adjusted learning rate to improve convergence. The learning rate used was 1e-3, with the exponential decay rate of 0.9 and 0.999 for the 1st and 2nd moment estimates respectively.

A more detailed description of these enhancements for the proposed architecture can be found in the Supplementary Methods.

#### Training methodology

The Enhanced model leverages regularization capabilities of the Baseline model. By observing the model learning trajectory, it was discovered that the validation loss never increased during training but rather converges to a value with minor fluctuations. The validation set then no longer serves the purpose for model checkpointing. Thus, a better usage of validation data is to add it back to the training dataset. The training process was stopped once the training loss plateaued, the model checkpoint being the highest training metric value, Area Under the (Receiver Operating Characteristic) Curve (AUC).

The dataset that was used for testing contained over 16 M datapoints. With such a large evaluation dataset, the simplest evaluation strategy as a single train-test split was preferred to cross-fold validation. The main purpose of the evaluation dataset is to test the hypotheses that are stated in the study, to reach a statistically significant conclusion when comparing results.

A batch size of 128 was used, along with batch normalisation and the ReLU activation function, see Fig. 1. The binary cross-entropy loss function was used and it was weighted to take account of the imbalance between the seizure and non-seizure target classes.

During training both the Enhanced and Baseline models were trained with three different seeds. During inference, the probabilistic outputs of the three models are averaged. This practice is common in machine learning, e.g. Kaggle competitions, to reduce the variability of the results to the random seed (weight initialization, data order during training, etc.) while increasing the sensitivity of the performance to the architecture changes.

### Pseudo labelling for site-specific adaptation

Distribution shift is a well-known challenge in ML. This is where the performance of a model trained on data from one set of conditions degrades when it is tested on a target dataset produced under a different set of conditions. This performance reduction due to distribution shift has been observed in neonatal seizure detection^19,23,41,46^. This hinders the usefulness of public and commercial off-the-shelf models.

Distribution shift in neonatal seizure detection can occur between recording sites due firstly to differences in recording procedures and methods such as: electrode application methods, types of electrodes used, filters used in recording equipment, sampling rate, and recording procedures. Secondly it can be due to differences in expert annotators regarding the event instance and even more so regarding its onset and offset times^47^. The first set of causes are reducible while the second set are not.

Here the distribution shift is addressed by using a pseudo labelling method. Pseudo labelling is usually used in ML as part of Knowledge Distillation, where a large model or ensemble of large models is used as a teacher model^48^. This teacher model is used to create soft labels, i.e. class probabilities for an unlabelled dataset. These soft labels are then used by a much smaller student model as a training set and thus the student model both encapsulates the knowledge of the much larger model and achieves comparable performance results.

**Fig. 2:**
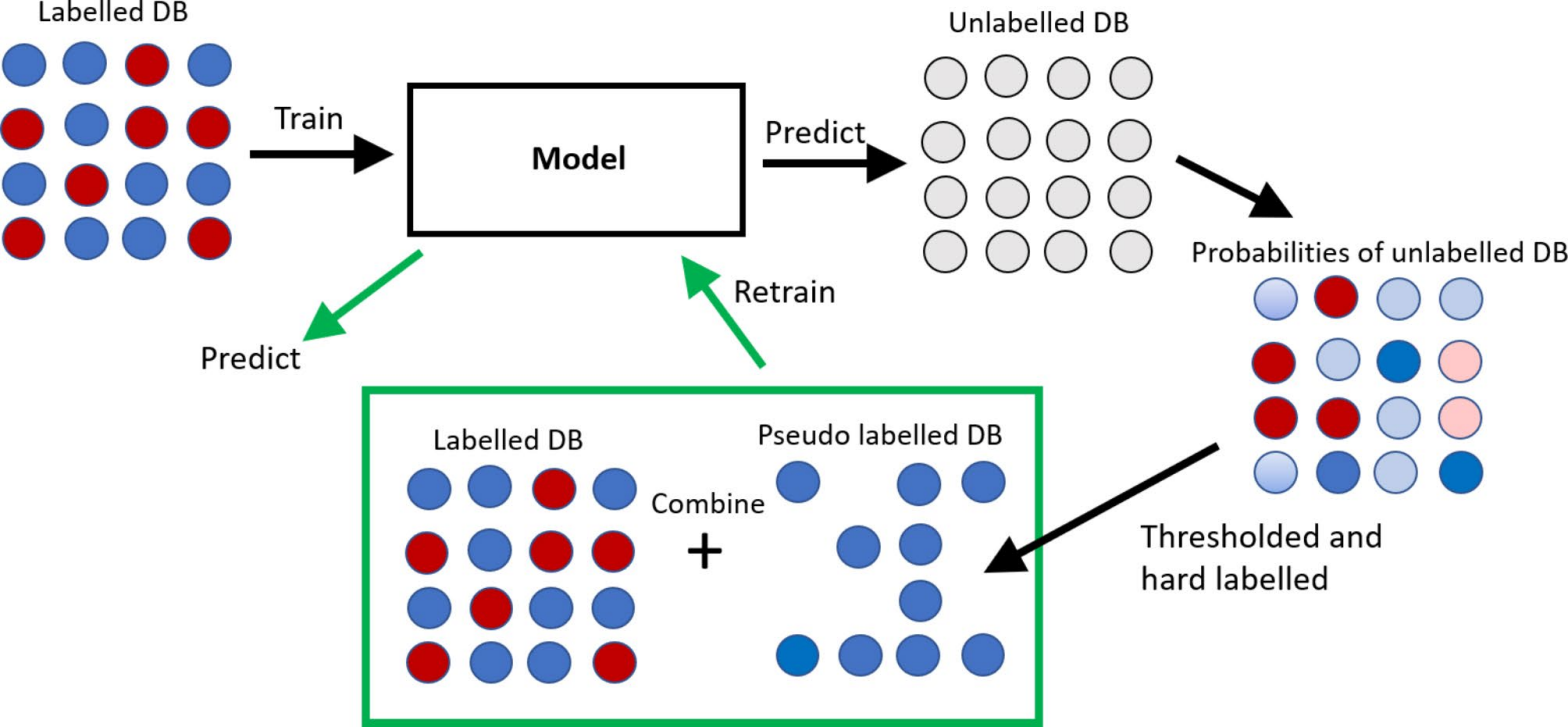
The pseudo labelling technique trains a model using a labelled database (DB) from Site A that generates probabilities of seizure/non-seizure for an unlabelled DB from Site B. Using a thresholding method the probabilities are hard labelled and so this pseudo labelled DB encapsulates information from Site B. This pseudo labelled DB is combined with the original labelled DB to form a larger training DB. The model is retrained and thus adapted to Site B specific conditions.

In this research we employ a pseudo labelling technique where the teacher and student models are the same. The Enhanced model is initially trained on the publicly available labelled dataset *SmallDB*^11^, which is detailed below. This is then used as a teacher model to create soft labels, i.e. probabilities for the unlabelled dataset from target conditions, *SampleDB*, which is also detailed below. *SampleDB* is not annotated for seizures while authors claim some may be present^12^. Thus, we decided to use this dataset to represent the non-seizure class only. Once the teacher model is run over *SampleDB*, the obtained probabilities are thresholded to create binary pseudo-labels – all probabilities less than a set threshold are converted to 0 to represent the non-seizure activity of the targeted condition. The threshold was chosen to discard 1.5% of the dataset as uncertain to avoid marking any potential seizure activity as non-seizure. The new pseudo-labelled dataset, which encapsulates non-seizure information from target conditions, was then combined with the labelled dataset *SmallDB* to form a larger training dataset on which the model was completely retrained to address the distribution shift. The process is depicted conceptually in Fig. 2. It should be noted that if both classes can be confidently represented in a sample dataset, then fine-tuning of the model rather than a complete retraining is advisable.

In contrast to Knowledge Distillation, the teacher and student models here are the same and the soft labels cannot be used. If the soft labels are used as pseudo labels, then there would be no extra information added when the model is retrained or finetuned as the probabilities generated by the model would be the same as the soft labels; there would be no difference, no error, no gradient flow, and no update to the weights. For this reason, the confidence-based hard labelling of a portion of *SampleDB* adds extra information from target conditions to the process and so mitigates the distribution shift. The process described above and utilized in this work provides an intuition into the pseudo-labelling methodology while the exact implementation can be different from case to case depending on the properties of a sample database.

### Datasets

To assess the impact of the size of the training dataset on the performance levels, the following data were utilized.

#### *TestDB* – the test dataset

For all testing purposes throughout the study, the same proprietary dataset is used (*TestDB*). It consists of continuous EEG recordings from 78 neonates, with 23 experiencing seizure events, recorded at the Neonatal Intensive Care Unit (NICU) of Cork University Maternity Hospital (CUMH). This dataset comprises of 4,570 h of unedited EEG recordings, with 57.7 h of seizure activity, 1.3% of the dataset, from 1,704 seizure events. The eight-channel bipolar montage (F4-C4, C4-O2, F3-C3, C3-O1, T4-C4, C4-Cz, Cz-C3, C3-T3) was used and the recordings were performed at either 256 Hz or 200 Hz. The database was previously used as a test dataset in^30^. Such a large and diverse dataset where no data pre-selection, editing or artefact rejection or removal was conducted, is needed for testing purposes to assure sufficient robustness and sensitivity to the changes of the architecture or training data.

#### Training datasets

Two different datasets are used for training purposes. The first dataset (*SmallDB*) is a publicly available database^11^. This dataset consists of short 1–2 h excerpts from 79 neonates who were admitted to the Helsinki University Hospital. This dataset comprises of 112 h of EEG recordings with 11 h of seizure activity, 9.8% of the dataset, from 342 seizure events. Eighteen channels of EEG were recorded at 256 Hz. The dataset is further detailed and analysed in^20^ with benchmark performances reported.

The second dataset used for training (*LargeDB*) consists of continuous EEG recording of 72 neonates taken at the CUMH NICU. Of the 72 neonates 18 experienced seizures from hypoxic ischemic encephalopathy (HIE) brain injury. The dataset for these 18 neonates consists of 834 h of continuous multichannel EEG recordings, with a total of 77.7 h of annotated seizure activity, 8.7% of the dataset, from 1,389 seizure events. One hour of background activity was included from each of the remaining 54 non-seizure patients. The total length of the dataset is 889 h. The same dataset was also used in other studies^17,19,30^, and for the Baseline model^23^. This dataset is 8 times larger and much more diverse in background neonatal EEG activity than *SmallDB* due to the continuous recording setup and no artefact removal. In addition, this dataset shares the same recording conditions as the test dataset. The number of datapoints used in training and testing are given in Supplementary Table S1.

#### *SampleDB* – unlabelled sample from test conditions used for site-specific adaptation

For pseudo-labelling purposes, a sample of the targeted testing conditions is required. For this another publicly available dataset is utilized^12^. This dataset (*SampleDB*) consists of short 1-hour EEG recordings also taken in CUMH NICU. *SampleDB* contains 53 neonates who were diagnosed with HIE, and totals 169 h. This dataset is not annotated for seizures events.

### Performance Metrics

#### AUC and AUC90

A variety of performance metrics are used in this research to give a full performance comparison^49^. Area Under the (Receiver Operating Characteristic (ROC)) Curve (AUC) is used as a performance metric. This measures the area under the curve when the sensitivity of a binary classifier is plotted against the specificity (or 1- specificity) at various threshold levels. Sensitivity is the percentage of seizure datapoints correctly labelled as seizure by the model; specificity is the percentage of non-seizure datapoints correctly labelled as non-seizure by the model. AUC is the most commonly used metric to compare the performances of seizure detection algorithms^49^ irrespective of the chosen operating point. The AUC90 metric calculates the area with Specificity > 90%. AUC90 is more representative of the performance in a clinical setting where a low number of false detections per hour is preferable. In this research AUC is reported as 0 to 1 or as 0 to 100%, i.e. % of the optimal 1.0 of AUC.

In order to determine if the differences in AUC are statistically significant, the relationship between AUC and the Wilcoxon test is utilized^50,51^. The standard error for these tests is approximately inversely proportional to the square root of the product of the no. of datapoints in each target class for the test dataset. As *TestDB* has a very large no. of datapoints (270,723/16,244,265 for seizure/non-seizure target classes), this standard error is very large which is the primary reason that all the differences in AUC in this manuscript are statistically significant. Further details of the statistical tests and the number of datapoints for each dataset are given in the Supplementary Information.

The relative error rate reduction percentage is given for comparison purposes, which gives the percentage of the total possible error reduction that is achieved, e.g. if an AUC of 0.96 is increased to an AUC of 0.98, this represents a 50% relative error rate reduction ((1-0.98) –(1- 0.96))/(1.0–0.96).

#### Precision-recall curve

As the test dataset, *TestDB*, has a large class imbalance the precision-recall curve is also given as a performance metric. Precision is the percentage of datapoints labelled as seizure by the model that are actual seizures, recall is another name for sensitivity, the percentage of seizure datapoints correctly labelled as seizure by the model. Thus, the precision-recall curve gives the trade-off between these two metrics at different threshold levels. The Area Under the Precision-Recall Curve (AUC-PR) is also given as an overall metric.

#### Event metrics – false detections per hour and good detection rate

While AUC is a comprehensive performance metric that measures the discriminative capacity of a model to separate seizure datapoints from non-seizure datapoints, the event metrics^49^ have a clearer clinical meaning and are more intuitive to interpret. The event metrics are the percentage of seizure events detected (any overlap with the ground truth) and the number of false seizure alarms per hour. One can plot a curve of good (event) detection rate vs. the number of false detections per hour, which offers a clear picture of the trade-off between correctly capturing as many seizure events as possible and the corresponding levels of false alarms.

## Results

**Fig. 3:**
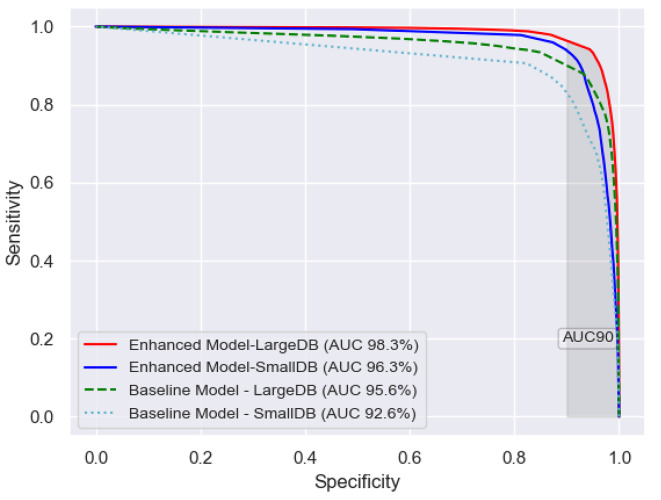
Comparison of ROC curves and AUC for the four experimental scenarios to assess the contribution of technical know-how versus that of data. The Baseline and Enhanced Models both use 2 different training dataset scenarios, *SmallDB* and *LargeDB*, *TestDB* being the test dataset in all cases. The AUC90 area, shaded in grey, is also given. More technical know-how is more important than more data in these scenarios while the combination of both gives the highest result. These differences in AUC are all statistically significant – see Supplementary Information for further information.

Tables 1 and 2; Fig. 3 compare the effect of the performance improvement in AUC due to advances in technical know-how (Enhanced model vs. Baseline) compared to that obtained from having access to a larger training dataset (*SmallDB* vs. *LargeDB*). The test dataset in all cases was the same. The Baseline and the Enhanced models were trained both on *SmallDB* and *LargeDB.* In Table 1 the gains in performance, using the relative error rate reduction, from having access to a larger training dataset were 38% and 54%, the AUC percentage improving from 92.6 to 95.4 and from 96.3 to 98.3, respectively. Similarly, having *SmallDB/LargeDB* as training datasets but enhancing the architecture and training procedures of the model, the performance improved from 92.6 to 96.3 and from 95.4 to 98.3, which correspond to 50% and 63% relative error rate reduction, respectively. The largest performance improvement comes from combining both having access to a larger training dataset and enhancing the architecture and training procedures of the model, the performance improved from 92.6 to 98.3, which corresponds to a 77% relative error rate reduction.

**Table 1:** Comparison of four experimental scenarios to assess the contribution of technical know-how versus that of data, using AUC. The Baseline and Enhanced Models both use 2 different training dataset scenarios, *SmallDB* and *LargeDB*, *TestDB* being the test dataset in all cases. The relative error reduction rate in AUC is also given. More technical know-how is more important than more data in these scenarios while the combination of both gives the highest result. These differences in AUC are all statistically significant – see Supplementary Information for further information.

		
AUC	*SmallDB*	*LargeDB*	Relative error rate reduction %
Baseline Model	92.6%	95.4%	38%
Enhanced Model	96.3%	98.3%	54%
Relative error rate reduction	50%	63%	77%

**Table 2:** Comparison of four experimental scenarios to assess the contribution of technical know-how versus data using AUC90. The Baseline and Enhanced Models both use 2 different training dataset scenarios, *SmallDB* and the *LargeDB*, *TestDB* being the test dataset in all cases. The relative error reduction rate in AUC90 is also given. More data is more important than more technical know-how in these scenarios while the best results are achieved by combining both.

		
AUC	*SmallDB*	*LargeDB*	Relative error rate reduction
Baseline Model	62.8%	79.0%	44%
Enhanced Model	72.3%	86.7%	52%
Relative error rate reduction	26%	36%	64%

Similar to Table 1, which uses AUC, Table 2 uses the AUC90 metric to compare the effect of the performance improvement due to advances in technical know-how compared to that obtained from having access to a larger training dataset. The test dataset is the same (*TestDB*) and the Baseline and the Enhanced models were trained both on *SmallDB* and *LargeDB.* The relative error rate reduction gains in performance from having access to a larger training dataset were 44% and 52%, the AUC90% improving from 62.8 to 79.0 and from 72.3 to 86.7, respectively. Similarly, having *SmallDB/LargeDB* as training datasets but enhancing the architecture and training procedures of the model, the performance improved from 62.8 to 72.3 and from 79.0 to 86.7, which correspond to 26% and 36% relative error rate reduction, respectively. Similar to the AUC the largest performance improvement comes from combining both having access to a larger training dataset and enhancing the architecture and training procedures of the model, the performance improved from 62.8 to 86.7, which corresponds to a 64% relative error rate reduction.

Figure 3 gives the ROC curves for the four experimental scenarios, the Baseline and the Enhanced models were both trained on *SmallDB* and *LargeDB*, the test dataset is the same (*TestDB*). These curves compare the performance improvement due to adding more data to that of employing more technical know-how along different threshold levels. Further the trade-off between sensitivity and specificity can be seen across the four experimental scenarios. As seen also in Tables 1 and 2 adding more data increases the ROC-AUC more inside the AUC90 area than outside (Baseline Model-*SmallDB* to Baseline Model-*LargeDB* and Enhanced Model-*SmallDB* to Baseline Model-*LargeDB*). Also as seen in Tables 1 and 2 adding more technical know-how contributes more outside the AUC90 area than inside (Baseline Model-*SmallDB* to Enhanced Model-*SmallDB* and Baseline Model-*LargeDB* to Enhanced Model-*LargeDB*). Adding more data and more technical know-how, Enhanced Model-*LargeDB*, outperforms the other three experimental scenarios.

**Fig. 4:**
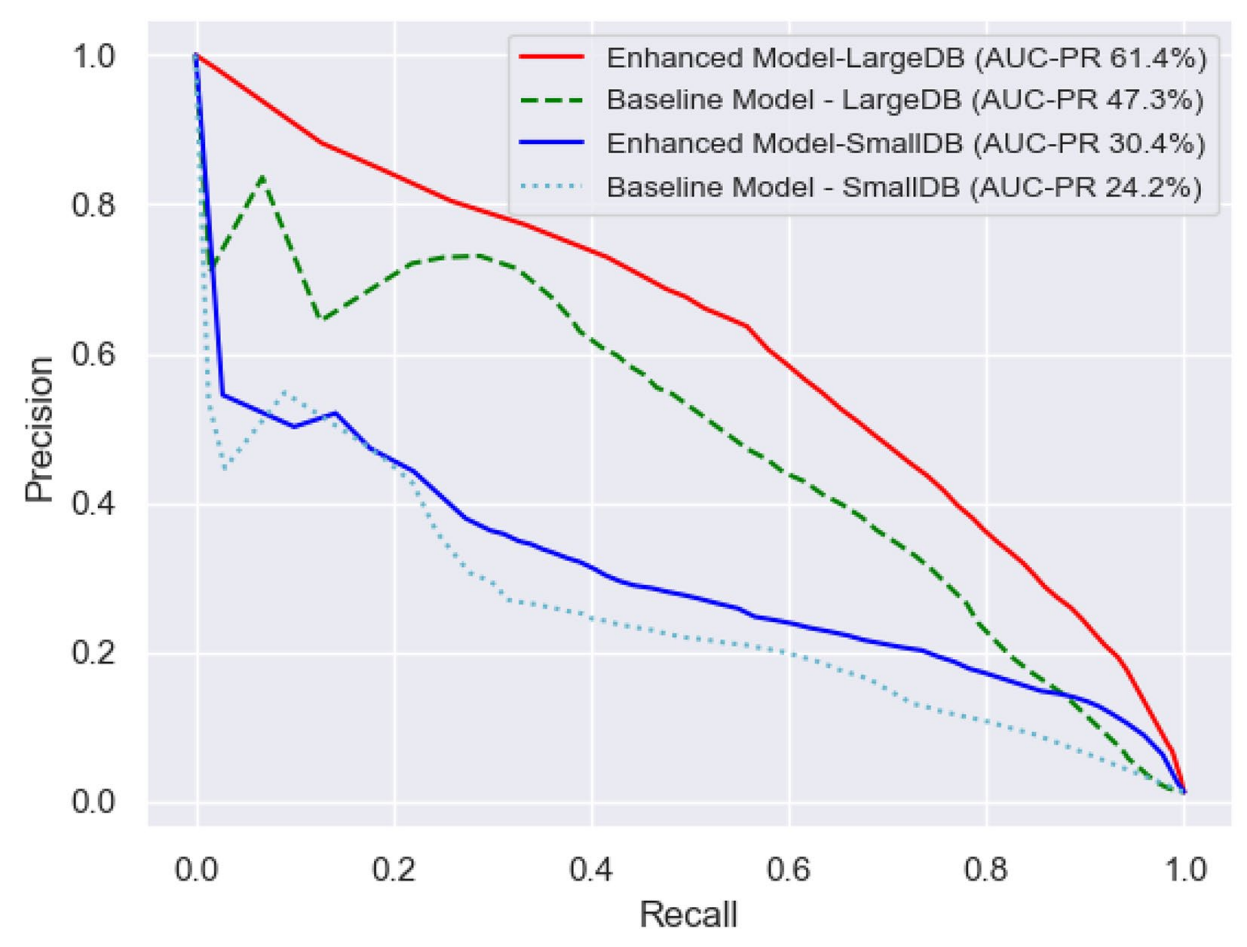
Comparison of precision-recall curves of the four experimental scenarios to assess the contribution of technical know-how versus that of data. The Baseline and Enhanced Models both use 2 different training dataset scenarios, *SmallDB* and *LargeDB*, *TestDB* being the test dataset in all cases. The areas under the curves (AUC-PR) are also given. More data has a larger impact here than more technical know-how with the combination of both giving the best performance. The positive class is seizure.

The precision-recall curves and the AUC-PR for the four experimental scenarios are given in Fig. 4. Again, the Baseline and Enhanced Models both use 2 different training dataset scenarios, *SmallDB* and *LargeDB*, *TestDB* being the test dataset in all cases. The trade-off between precision and recall along different operating threshold levels can be seen across the four experimental scenarios. These curves also compare the performance improvement due to adding more data to that of employing more technical know-how. Having access to a larger training dataset increases the AUC-PR percentage from 24.2 to 47.3, i.e. by 23.1, for the Baseline Model and from 30.4 to 61.4, i.e. by 31.0, for the Enhanced model. In contrast employing the latest technical know-how and training procedures on the training dataset *SmallDB*, increases the AUC-PR percentage from 24.2 to 30.4, i.e. by 6.2 and on the training dataset *LargeDB*, AUC-PR percentage increases from 47.3 to 61.4, i.e. by 14.1. Thus in terms of the AUC-PR, adding more data is more beneficial than adding more technical know-how but the combination of both, adding more data and more technical know-how, Enhanced Model-*LargeDB*, again outperforms the other three scenarios, with an AUC-PR percentage of 61.4.

Table 3 compares the performance of the Enhanced model to that of Public SVM. Both models were trained on the publicly available *SmallDB*. Because the Public SVM utilizes a computationally heavy feature extraction procedure, the test dataset here is a random sample of 5 seizure patients from *TestDB*, comprising of 410 hours of total recording, 4.8 hours of seizures with 70 individual seizure events. It can be seen that the Enhanced model outperforms the Public SVM with an AUC of 97.7% compared to an AUC of 89.7%. This represents a relative error rate reduction of 78% and 48% for AUC and AUC90, respectively. Table 3 also gives the comparison of the inference run-time between the Public SVM and the Enhanced model. In both cases, the same hardware was utilized without any multi-processing or multi-threading capabilities enabled for the inference (CPU only, Intel(R) Xeon(R) Gold 6138 CPU @ 2.00GHz, 10 Cores). It takes close to 2h to process 1h of EEG for the public SVM whereas the Enhanced model only takes 6 minutes to do the same job.

**Table 3:** The performance of Enhanced model (C) versus the current public benchmark, the Public SVM (the model weights are taken from web). Both models were trained on *SmallDB*. The test set used is a random subset of *TestDB* (410 hours). The inference time for both models is given.

	AUC	AUC90	Inference time (hours)
Public SVM	89.7%	71.3%	779.4
Enhanced Model	97.7%	84.9%	4.5

**Table 4:** Assessing the performance of the Enhanced mode (C) after distribution shift correction with pseudo-labelling. Model C is retrained by adding a sample of pseudo labelled data from target conditions to the training dataset (*SampleDB+SmallDB*). The increase in AUC is statistically significant – further details are given in Supplementary Information.

	AUC	AUC90
Enhanced model (Model C)	96.3%	72.3%
Model C + pseudo labelling	96.8%	78.3%

The architecture, code and weights of the Enhanced model trained on *SmallDB* are made publicly available to form a new public benchmark for subsequent comparisons in the area. This new model is both more accurate at detecting seizures and has a much lower computational load than the current publicly available algorithm.

**Fig. 5:**
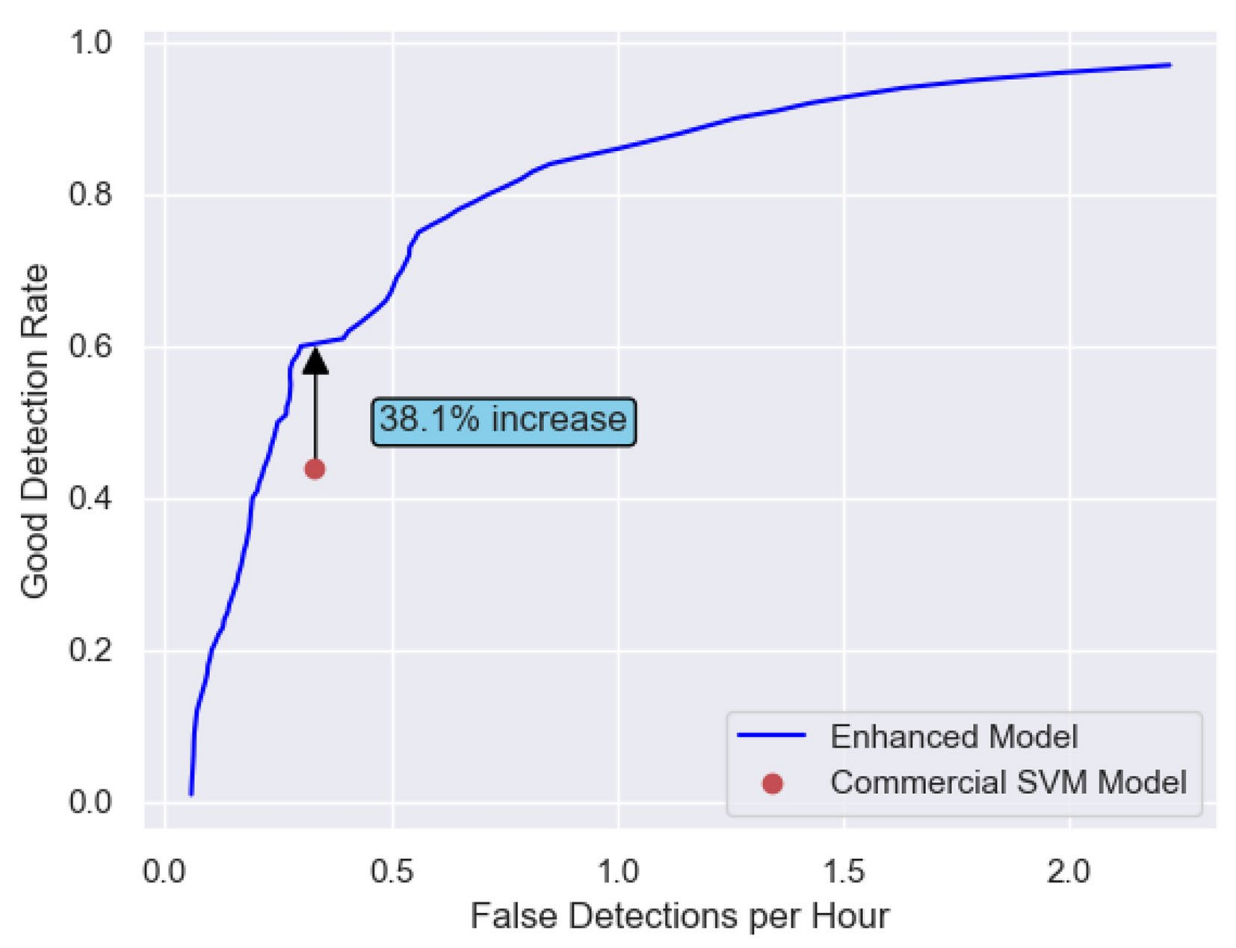
Comparison of the Enhanced model (model C) with the Commercial SVM model using the false detections per hour versus the good detection rate. These event metrics are calculated by obtaining the average of the event metrics across the 23 patients from *TestDB* who experienced seizures. The Commercial SVM model utilizes a predefined threshold which results in a single operating point.

Figure 5 compares the performance of the Enhanced model trained on *SmallDB* with the Commercial SVM model^17,19,39^ using the event metrics, i.e. the false detections per hour versus the good detection rate. The event metrics are calculated by obtaining the average of the event metrics across the 23 patients who experienced seizures from *TestDB*. The Commercial SVM model utilizes a predefined threshold which results in a single operating point. It can be seen the SVM operating point is below the operative curve of the Enhanced model. At a rate of 0.33 FDs/h the commercial SVM model obtains a good detection rate of 43.6% whereas the Enhanced model achieves 60.3%. This represents a 38% relative improvement in the good detection rate at the same fixed rate of false detections.

Table 4 shows the results of adapting the newly publicly available Enhanced model (model C) to testing conditions which are different from the data on which the model was trained (*SmallDB*). Pseudo-labelling a sample of the targeted condition (*SampleDB*) and retraining the model on (*SmallDB + SampleDB*), the AUC and AUC90 were further improved from 96.3 to 96.8% and from 72.3 to 78.3%, respectively. The weights of this model are also made publicly.

## Discussion

In many areas of applied ML it is believed that data is the main and at times the only ingredient to successful ML-based solution^8,10,52,53^. This tends to arise from the fact that data is seen as the easiest, most straightforward, and guaranteed way to improve the performance whereas acquiring an additional ML expertise is tedious and requires substantial investment of resources without a guarantee of a return on investment. While the assumption regarding the data is in general valid, at least for data of good quality, acquiring more data can be expensive. Moreover, in classical areas of ML, such as NLP, computer vision, speech recognition, major advancements came from architectural and technical innovations, be it convolution architectures^1,3,4^, attention mechanisms^36,37,54^, reinforcement learning with human feedback^55^, etc. In those areas where a large stable amount of data exists the key focus for researchers is performance improvement from advances in technical know-how. A representative example is the ImageNet computer vision task where technical know-how and improved training regimes have led to increased performance^5,56^.

In this study, we contrast the benefits of technical know-how with the availability of a larger training dataset in the context of neonatal EEG analysis. Like many other medical domain areas neonatal seizure detection lacks publicly available datasets^11^, data collection is costly, the process of labelling is time-consuming and expensive since domain experts are required. Moreover, most large datasets are proprietary, and the technical progress is hindered by the lack of meaningful comparison. This study’s comparison will aid other researchers in deciding how to invest their time and resources, namely into acquiring more data and/or technical ML expertise.

### Are data all you need?

This study has found that even with limited data at hand a competitive performance can be obtained if the latest architectural and training enhancements are incorporated, as seen in Table 1; Fig. 3. Using even an 8-times larger dataset with many more annotated seizures and much more diverse background activity, the performance gains in AUC are smaller than using the smaller training dataset with the Enhanced model.

On the other hand, the gains from getting more data are also evident. Both Table 1; Fig. 3 indicate that while getting more data is not as beneficial as finding the right representation and training method (for the considered setup), extra data result in better performance in all experiments. In fact, the best performing model is obtained when combining the larger training dataset (*LargeDB*) and all considered enhancements, reaching an AUC of 98.3%, which represents a 77% relative error rate reduction over the 92.6% with the Baseline model and publicly available training dataset (*SmallDB*).

Further using AUC90 as the performance metric Table 2 also shows that adding more deep learning enhancements improves the performance considerably but, unlike AUC, adding more training data results in even better performance improvement. This highlights the fact that relative importance of adding more data compared with adding more technical advancements can change at different portions of the Receiver Operating Characteristic Curve, and consequently at different threshold operating points of the model.

Due to the class imbalance the trade-off between precision and recall at different threshold operating points is given in Fig. 4. Here the AUC-PR of the four experimental scenarios show that the performance improvement that can be achieved from adding more data is more beneficial that adding more technical enhancements, with the best performance again is achieved by combining both which gives an AUC-PR of 61.4%.

Thus, different metrics offer differing perspectives on whether technical know-how is more beneficial than having access to a larger, more diverse training dataset. Using AUC as a metric, the performance improvements from technical know-how are larger than those that can be achieved by having access to a larger, more diverse training dataset. In contrast using AUC90 and AUC-PR as metrics, it is shown that having access to a larger, more diverse training dataset is more beneficial compared to using the latest technical know-how. For all metrics, for a given dataset it is shown that using the latest architectural and technical advancements leads to performance improvements. These important findings and the contrasting suite of metrics will aid practitioners to evaluate the cost of acquiring extra data vs. the cost of acquiring practical ML expertise. This advocates for further architectural research in the area of applied deep learning methods and where possible further dataset acquisition, especially in domain areas with small publicly available datasets.

Since *SmallDB* has been published in 2019 it has been used extensively as a training/testing dataset by many researchers in neonatal seizure detection^23–25,28,30,32^. In this research we have used *SmallDB* as a baseline training dataset. Even though using *SmallDB* as a training dataset for the Enhanced model gives an AUC of 96.3% which is high in absolute terms, in our experience for a model to be usable in a clinical setting it is also instructive to investigate the event metrics and the precision-recall curve. As can be seen in Fig. 5, using the event metrics the Enhanced model outperforms a model that is currently been used in a clinical setting, the Commercial SVM model, yet it still has further scope for improvement. Similarly, the precision-recall curves given in Fig. 4 also show that there is further scope for improvement in the detection of seizures and the level of false detections. For these reasons and due to it being publicly available we considered this relatively small dataset, *SmallDB*, as a baseline training dataset in our neonatal seizure detection research.

We have not investigated comprehensively the effect on performance of progressively adding more data from a small base to the training set and comparing this to the incremental adding of more technical improvements. For example, if at a certain level of data there is a ceiling effect, beyond which more performance improvement can be achieved from technical improvements rather than adding training data. What is noteworthy and novel about this research is showing the comparison of the scale of improvement due to increasing the size of the training dataset from a small and limited publicly available dataset to an 8-times larger dataset of continuous EEG recording, to the scale of improvement due to adding more technical know-how. It also shows how much performance improvement can be extracted from training on this publicly available dataset using the very latest of deep learning techniques.

### A new benchmark

The Enhanced model trained on *SmallDB* shows a significant improvement over the publicly available (SVM) seizure detection model, both in terms of performance and computational load. The Enhanced model avoids the use of hand-crafted features instead learning the EEG signal characteristics and patterns of neonatal seizures directly from the EEG signal. While it would be expected that a deep learning model would outperform an SVM model, this SVM is the current public benchmark and so the improvements in performance and computational load for the proposed new public benchmark are noteworthy.

The proposed benchmark model which is trained on *SmallDB* also outperforms the commercially available SVM model by 38.1% in the good detection rate at a desired false alarm rate of 0.33 FDs/h, as seen in Fig. 5. The commercial SVM model was developed in 2013^19^. Here also, while a deep learning model is expected to outperform an SVM model, this SVM model is in current clinical use and so is an important comparison. This highlights the fast pace at which new and better solutions come into existence and the scale of performance improvement that can be expected from these solutions over the models that are in current clinical use. The performance improvement of the benchmark model is further noteworthy as it was trained on the *SmallDB*, showing how much seizure detection capabilities can be extracted from this relatively small publicly available dataset by harnessing the latest in deep learning techniques.

On many occasions, privately held large proprietary datasets cannot be made public for ethical reasons. As with other fields in computational medical research neonatal seizure detection suffers from the fundamental problem that it is practically impossible to compare or reproduce the results of other groups. It is difficult to assess where a genuine technical contribution was made and if better results were obtained due to data related manipulations such as a larger dataset used for training or an easier dataset used for testing. In addition, only centres that have access to data can benefit from ML models and their usage in clinical settings. Publicly available models become as important as publicly available datasets. The model and code that are released in this study can be utilized for comparative assessment on privately held datasets and for clinical purposes once satisfactory levels of performance are observed by practitioners. This model will enable verifiable, timely and comprehensively peer-reviewed improvements in this important area of research. The Enhanced model outperforms the Baseline model with relative error rate reductions in AUC of 50% and 63% using *SmallDB* and *LargeDB* as the training sets, respectively. Using AUC90 and AUC-PR as metrics the Enhanced model also shows performance gains over the Baseline model using both training datasets, although not as large as with the AUC metric. The incorporation of the latest advancements in architecture and training methodology allowed for improvement of data representations by increasing the network depth and the receptive field from 5s to 10s, and the input size from 8s to 16s – something that was shown to be ineffective for the Baseline model^23^. This change allowed for an improved ability to encode the characteristics of longer evolving rhythmic patterns of seizures. The Enhanced model with the larger input size of 16s relies less on the post processing while bringing more of the decision-making capabilities of the network into the end-to-end optimization routine.

### Adapting the benchmark model to target conditions

The test conditions where the public model can be deployed can significantly differ from those on which the model was trained. The differences may come from sensors specification, clinical preparation and application procedures, acquisition hardware and software. A sample data from the targeted conditions can usually be sourced. In this work we show how such sample data without any expert annotations required can be used to address the distribution shift and improve the performance of the off-the-shelf pretrained publicly available model with the help of pseudo-labelling.

This process can be adopted to address the distribution shift in other domain areas and for other models as unannotated dataset are often available or inexpensive to collate. This method can also be used to retrain/fine-tune other models to site-specific conditions.

Due to the fact that a relatively small dataset was pseudo labelled, *SampleDB* (169 h), compared to the much larger test dataset *TestDB* (4,570 h) and only the non-seizure target class was pseudo labelled, the gains in performance in AUC from this method are relatively small, 0.5%, but are statistically significant – see the Supplementary Information. These gains, the authors believe, should increase with a larger pseudo labelled dataset from target conditions and with more representations from both target classes.

The datasets which were used in this experiment, *SmallDB* and *SampleDB*, come from 2 different centres. The resultant model, whose code and weights are being publicly released, has an added element of reliability, robustness and generalisability since two different montages, signal conditions and acquisition procedures have been employed to record the data.

## Conclusion

A new state-of-the-art model for neonatal seizure detection has been introduced. The latest advancement in architecture and training procedures have been utilized. Using a publicly available dataset as a baseline we compared the performance improvements achieved from harnessing these latest advancements in technical know-how to those that can be achieved from having access to a larger and more diverse training dataset. It is shown that harnessing the latest in technical innovations and training and using a larger more diverse training dataset are both key ingredients in improving performance. Different metrics give differing perspectives with AUC showing that harnessing the latest in technical innovations and training regimes is more beneficial whereas AUC90 or AUC-PR show that using a larger more diverse training dataset is more beneficial. What is also noteworthy is that for a fixed dataset employing the latest technical and training innovations results in an improved performance in all metrics. Additionally, superior performance improvement in all scenarios is achieved by combining the latest technical innovations and training regimes with the larger more diverse training dataset. Furthermore, the new Enhanced model significantly surpasses in performance the current state-of-the-art deep learning model, a publicly available machine learning model and a commercial model. This demonstrates how much seizure detection capabilities can be extracted from the single publicly available dataset using the latest in deep learning techniques. The code and weights of the new state-of-the-art model are made publicly available. A new procedure to adjust this model to any targeted testing condition using pseudo-labelling has also been outlined.

## Electronic supplementary material

Below is the link to the electronic supplementary material.

Supplementary Material 1

## Data Availability

Two of the datasets analysed in this study, are publicly available through the Zenodo repository under the creative commons license by the: • Helsinki University Hospital, Finland, (*SmallDB*) available at https://zenodo.org/record/4940267. • INFANT Research Centre, University College Cork, Ireland, (*SampleDB)* available at https://zenodo.org/record/747757. The code and weights for the Enhanced model are available https://github.com/CiallC/Neonatal_seizure_detection_dl_algorithm.
