# Supplementary material for: Analysis of the impact of deep learning know-how and data in modelling neonatal EEG
Source: Sci Rep. 2024 Nov 14;14:28059. doi: 10.1038/s41598-024-78979-y (PMC11564755; doi:10.1038/s41598-024-78979-y)
Supplement: Supplementary file 1 — Supplementary Material 1 [file 41598_2024_78979_MOESM1_ESM.pdf]

Analysis of the impact of deep learning know-how and data in modelling neonatal EEG

Aengus Daly[1,2,3], Gordon Lightbody[2,3], Andriy Temko[2]
[1] Department of Mathematics, Munster Technological University, Ireland
[2] Department of Electrical and Electronic Engineering, University College Cork, Ireland
[3] INFANT Research Centre, University College Cork, Cork, Ireland
*aengus.daly@mtu.ie

Abstract

The performance gains achieved by deep learning models nowadays are mainly attributed to the usage of ever larger datasets. In this study, we present and contrast the performance gains that can be achieved via accessing larger high-quality datasets versus the gains that can be achieved from harnessing the latest deep learning architectural and training advances. Modelling neonatal EEG is particularly affected by the lack of publicly available large datasets. It is shown that greater performance gains can be achieved from harnessing the latest deep learning advances than using a larger training dataset when adopting AUC as a metric, whereas using AUC90 or AUC-PR as metrics greater performance gains are achieved from using a larger dataset than harnessing the latest deep learning advances. In all scenarios the best performance is obtained by combining both deep learning advances and larger datasets. A novel developed architecture is presented that outperforms the current state-of-the-art model for the task of neonatal seizure detection. A novel method to fine-tune the presented model towards site-specific settings based on pseudo labelling is also outlined. The code and the weights of the model are made publicly available for benchmarking future model performances for neonatal seizure detection.

Supplementary Methods

This section gives detailed descriptions and explanations of the key methodological advances used in this work, which are:

- the architectural innovations (residual connections, depthwise convolutions)
- data augmentation techniques (Mixup)
- the advances in the training routine (RAdam optimizer)

It also details the statistical significance tests for the differences in AUC and the number of datapoints used during training and testing.

Architectural innovations

Residual connections

The Enhanced model incorporates residual/skip connections [1] that allow the network to learn improved data representations as the depth of the network increases, a capability the Baseline model did not possess [2].

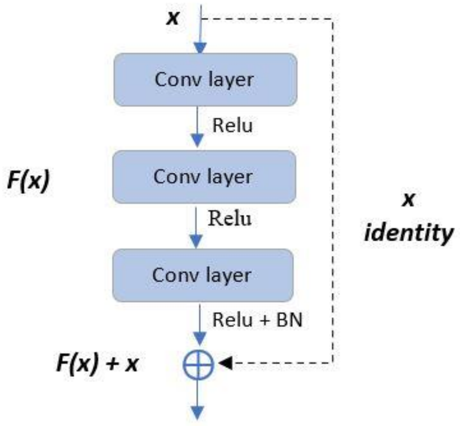

Figure S1. Residual connection (black dashed line) in a Feature Extraction Block (FEB), in the Enhanced model.

These residual/skip connections in the Enhanced model enable each Feature Extraction Block (FEB) to learn a residual mapping, see Figure S1. Let *H(x)* be the desired mapping of the FEB and *x* be the input to the block. Let *F(x)* be the mapping of the block then *F(x):= H(x) - x*, i.e. the residual mapping equals the difference between the desired underlying mapping for the block , *H(x),* and the input to the block, *x*. *F(x) + x* equals the final mapping and it is much easier for the training routine to optimize this residual mapping rather than the mapping without a residual connection. This enhances the network's ability to learn data representations as the depth of the network increases and so improves the network's performance [1].

Depthwise convolutions and feature block enhancements

The Enhanced network employs depthwise convolutions in conjunction with residual connections which were utilized and made popular by Xception Net [3] and MobileNet [4]. Depthwise convolutions apply separate filters to each input. It consists of 3 steps:

- Split the input and filter into channels
- Convolve each input with the respective filter
- Stack the convolved outputs together

Depthwise convolutions are analogous to the weighted sum operation in self-attention [5]. This attention-like grouping and weightings of feature maps extracts added information at channel level at various layers of the network, enabling the network to learn more detailed data representations. Similar to Transformers where the Multi-Head Self Attention blocks are placed before the fully connected blocks here depthwise convolution are placed at the start of the feature extraction block, see Fig. 1.

The design of the depthwise FEBs of 64>64>32 feature maps, see Fig. 1, is an inverted bottleneck that is included in recent advanced ConvNets [6], [7]. This architecture widens the network at the start of these feature extraction blocks to learn more detailed data representations from the depthwise convolutions which are subsequently summarized through the down-sampling via the 1X1 convolution. Thus, these feature extraction blocks efficiently retain the key characteristics of the more detailed data representations from depthwise convolutions leading to improved data representations within the network.

A larger kernel size of (5,1), corresponding to (time dimension, channel dimension), for the depthwise convolutional layers, compared to kernels of (3,1) in the other convolution layers, was

shown to improve representations in the Swin Transformers [8] was also adopted in this research. These larger kernels add variety throughout the network thus improving the ability of the network to learn more diverse, robust and accurate data representations.

Data augmentation

The Enhanced model engages two data augmentations techniques on the full dataset during training. Firstly the amplitude is randomly altered/rescaled, by one of four equally possible transformations, namely increase the signal amplitude by a random number between 0.5 and 1.5, a vertical flip, a combination of the previous two transformations, or no alteration [9].

This amplitude rescaling is followed by Mixup [10]. During training within each batch (size 128) each EEG signal input and its corresponding label is paired at random with another EEG signal input and its label. A linear interpolation of each pair of inputs (x_i, x_j) and their corresponding labels (y_i, y_j) produces a new augmented signal input and label as follows:

$$\tilde{x} = \lambda x_i + (1 - \lambda) x_j$$
$$\tilde{y} = \lambda y_i + (1 - \lambda) y_j \qquad (1)$$

where the weight, λ, is chosen at random from a beta distribution, $\lambda \sim Beta(0.8, 0.8)$.

Both data augmentation techniques are implemented as an infinite data generator.

The combination of these data augmentation techniques adds an element of signal diversity and a small random amount of noise to the training dataset, thus acting both as a regularizer to prevent overfitting and also improving the optimisation of the model by adding signal and label variety during training.

Optimization innovations

The Rectified Adam (RAdam) optimizer [11] is used in training the Enhanced model. The Adam optimizer adaptively adjusts the learning rate using a warm-up heuristic [12], which was found to have a large variance initially due to using only a relatively small no. of samples which can lead to convergence to poor local minima. RAdam, a variant of Adam further adjusts the variance of the adaptive learning rate [11], thus consistently improving the performance and speed of the optimization training routine, and is very robust to changes in the initial learning rate. The learning rate used was 1e-3, with the exponential decay rate of 0.9 and 0.999 for the 1st and 2nd moment estimates respectively.

No. of datapoints for training and testing

Table S1 below gives the no. of datapoints (total, no. with seizures, no. without seizures and the percentage that contain seizures) of the training (*SmallDB* and *LargeDB*) and testing (*TestDB*) datasets, for the Enhanced model. The targets used in all cases are per second annotations thus they equal the no. of datapoints given in Table S1.

Table S1. The number of datapoints of the training (SmallDB, Large DB) and testing (TestDB) datasets for the Enhanced model, the % of datapoints that contain a seizure is also given.

Dataset	***SmallDB***	***LargeDB***	***TestDB***
Total no. of datapoints	403,185	3,200,385	16,451,985
No. without seizures	363,586	2,920,666	16,244,265
No. with seizures	39,599	279,719	207,720
% with seizure	9.8%	8.7%	1.3%

Statistical Tests of Significance for differences in AUC

In order to test if the differences in AUC are statistically significant the relationship of the Area under the ROC curve(AUC) to the Wilcoxon test is used [13], [14]. First, the standard error (SE) of an AUC can be calculated using the following formula [13]:

$$SE(\theta) = \sqrt{\frac{\theta(1-\theta)+(n_A-1)(Q_1-\theta^2)+(n_N-1)(Q_2-\theta^2)}{n_A n_N}} \quad (1)$$

here Θ is the AUC, $Q_1 = \Theta/(2 - \Theta)$ and $Q_2 = 2\Theta^2/(1 + \Theta)$, n_A and n_N are the numbers of seizure (abnormal) and non-seizure (normal) datapoints. Second, the statistical significance of the difference in AUC of two models using the same test dataset can be calculated by evaluating the following z statistic, according to the following formula [14]:

$$z = \frac{\theta_1-\theta_2}{\sqrt{SE(\theta_1)^2+SE(\theta_2)^2-2rSE(\theta_1)SE(\theta_2)}} \quad (2)$$

here Θ_1 and Θ_2 are the AUC's for model 1 and model 2, respectively; *r* represents the estimated correlation between the two ROC curves as outlined in [14]. The corresponding *p* values for the two-tailed tests of all the differences in AUC presented in this research are less than 0.001 and so they are all deemed statistically significant. The main reason for this is that the values of n_A and n_N in *TestDB* are extremely large (270,723/16,244,265 for the seizure/non-seizure target classes), so the standard errors for all AUC differences are very small. Further, all the estimated correlations *r* in equation (2) for all these difference in AUC are greater than 0.47 which increases the corresponding z statistic values and subsequently reduces the resultant *p* values.

References

1. He, K., Zhang, X., Ren, S. & Sun, J. Deep Residual Learning for Image Recognition. in *2016 IEEE Conference on Computer Vision and Pattern Recognition (CVPR)* 770–778 (2016). doi:10.1109/CVPR.2016.90.
2. O'Shea, A., Lightbody, G., Boylan, G. & Temko, A. Neonatal seizure detection from raw multi-channel EEG using a fully convolutional architecture. *Neural Networks* **123**, 12–25 (2019).
3. Chollet, F. Xception: Deep Learning with Depthwise Separable Convolutions. in 1800–1807 (IEEE Computer Society, 2017). doi:10.1109/CVPR.2017.195.
4. Howard, A. G. *et al.* MobileNets: Efficient Convolutional Neural Networks for Mobile Vision Applications. Preprint at http://arxiv.org/abs/1704.04861 (2017).
5. Liu, Z. *et al.* A ConvNet for the 2020s. in *2022 IEEE/CVF Conference on Computer Vision and Pattern Recognition (CVPR)* 11966–11976 (2022). doi:10.1109/CVPR52688.2022.01167.

6. Tan, M. & Le, Q. EfficientNet: Rethinking Model Scaling for Convolutional Neural Networks. in *Proceedings of the 36th International Conference on Machine Learning* 6105–6114 (PMLR, 2019).
7. Sandler, M., Howard, A., Zhu, M., Zhmoginov, A. & Chen, L.-C. MobileNetV2: Inverted Residuals and Linear Bottlenecks. in 4510–4520 (IEEE Computer Society, 2018). doi:10.1109/CVPR.2018.00474.
8. Liu, Z. *et al.* Swin Transformer: Hierarchical Vision Transformer using Shifted Windows. in 9992–10002 (IEEE Computer Society, 2021). doi:10.1109/ICCV48922.2021.00986.
9. Daly, A., O'Shea, A., Lightbody, G. & Temko, A. Towards Deeper Neural Networks for Neonatal Seizure Detection. in *2021 43rd Annual International Conference of the IEEE Engineering in Medicine Biology Society (EMBC)* 920–923 (2021). doi:10.1109/EMBC46164.2021.9629485.
10. Zhang, H., Cisse, M., Dauphin, Y. N. & Lopez-Paz, D. mixup: Beyond Empirical Risk Minimization. in (International Conference on Learning Representations, 2018). doi:10.48550/arXiv.1710.09412.
11. Liu, L. *et al.* On the Variance of the Adaptive Learning Rate and Beyond. in (International Conference on Learning Representations, 2020). doi:10.48550/arXiv.1908.03265.
12. Kingma, D. P. & Ba, J. Adam: A Method for Stochastic Optimization. in (International Conference for Learning Representations, San Diego, 2015). doi:org/10.48550/arXiv.1412.6980.
13. Hanley, J. A. & McNeil, B. J. The meaning and use of the area under a receiver operating characteristic (ROC) curve. *Radiology* **143**, 29–36 (1982).
14. Hanley, J. A. & McNeil, B. J. A method of comparing the areas under receiver operating characteristic curves derived from the same cases. *Radiology* **148**, 839–843 (1983).
